# 3D Off-Lattice Coarse-Grained Monte Carlo Simulations for Nucleation of Alkaline Aluminosilicate Gels

**DOI:** 10.3390/ma16051863

**Published:** 2023-02-24

**Authors:** Mohammadreza Izadifar, Nicolas Castrillon Valencia, Peng Xiao, Neven Ukrainczyk, Eduardus Koenders

**Affiliations:** Institute of Construction and Building Materials, Technical University of Darmstadt, Franziska-Braun-Str. 3, 64287 Darmstadt, Germany

**Keywords:** 3D off-lattice coarse-grained Monte Carlo, aluminosilicate geopolymer gels, metakaolinite-based geopolymer, alkali silicate solution, nucleation, nanostructure, cluster size distribution, pore size distribution

## Abstract

This work presents a 3D off-lattice coarse-grained Monte Carlo (CGMC) approach to simulate the nucleation of alkaline aluminosilicate gels, their nanostructure particle size, and their pore size distribution. In this model, four monomer species are coarse-grained with different particle sizes. The novelty is extending the previous on-lattice approach from White et al. (2012 and 2020) by implementing a full off-lattice numerical implementation to consider tetrahedral geometrical constraints when aggregating the particles into clusters. Aggregation of the dissolved silicate and aluminate monomers was simulated until reaching the equilibrium condition of 16.46% and 17.04% in particle number, respectively. The cluster size formation was analyzed as a function of iteration step evolution. The obtained equilibrated nano-structure was digitized to obtain the pore size distribution and this was compared with the on-lattice CGMC and measurement results from White et al. The observed difference highlighted the importance of the developed off-lattice CGMC approach to better describe the nanostructure of aluminosilicate gels.

## 1. Introduction

Geopolymer or alkaline aluminate silicate geopolymer material [1] is typically synthesized by metakaolinite [2] as an aluminosilicate powder mixed with potassium silicate or sodium silicate solution precursor [3,4,5]. The major role of silicate solutions is to trigger the process of alkalinization. In this dissolution–precipitation geopolymerization reaction, first, the bonds of the silicon and aluminum species from aluminosilicate powder solid material are broken by hydroxyl (OH^−^) of the alkaline solution, followed by a polycondensation reaction and the final formation of the aluminosilicate network [6], which comprises Al and Si tetrahedra [7] linked by oxygen bridging bonds [4,8]. Although geopolymer binders have been proven to have identical mechanical properties compared to ordinary Portland cement, approximately 80–90% less global anthropogenic CO_2_ emission in geopolymer productions make this material an environmentally friendly alternative to traditional cement [9]. Moreover, geopolymers exhibit superior acid resistance compared to Portland cement, which is considered to be one of the most frequently reported privileges of geopolymers [10,11,12,13,14].

The nanoscale, 10^−9^–10^−5^ m, is very significant to study because it is the first step in the upscaling effort to predict measurable materials’ properties. Nucleation or polymerization (cluster formation) is used as the initial component of the multiscale modeling [15,16], considering it as the first larger scale after atomistic simulations, 10^−8^–10^−7^ m, comprised of monomer species, e.g., atoms, ions, or molecules, to form a new thermodynamic configuration or structure at the atomic or molecular level [17]. Classical nucleation theory (CNT) describes the formation of a critical nuclei cluster size, i.e., a transition into a first stable solid that can grow further. The size of this nuclei is proportional to its surface energy and is inversely proportional to the supersaturation of the solution. However, the usage of CNT is not relevant for geopolymers due to the following reasons. First, CNT is challenged by obtaining the nuclei surface energy with good accuracy. Moreover, the system may find alternative routes around the high CNT energy barrier, by a series of metastable states [18], described by the non-classical crystallization (NCC) theory. Finally, and most importantly, CNT refers only to the direct formation of crystals, which is thus not relevant for the precipitation of amorphous aluminosilicate gels (i.e., geopolymers). Thus, Yang and White [19,20] proposed CGMC simulation, initially using a very simple on-lattice implementation, to describe the main mechanisms behind the geopolymerization reaction: dissolution, polycondensation, and (cluster) reorganization. Crystallization can be classified as either homogenous or heterogeneous. The aggregation is one of the major processes involving monomer contributions on the surface of the nuclei for the heterogeneous growth of aggregates [21,22]. In this work, inspired by Yang and White [19] and to simplify the simulation process, the activation energy is ignored. Only the binding energy part is kept, so this method pays more attention to the change in energy before and after the equilibrium states without considering the speed of energy change (reaction kinetics). White et al. [21] used quantum chemical-based interaction (dimerization) energies, determined through density functional theory computations, in an on-lattice coarse-grained Monte Carlo (CGMC) simulation of the initial stages of gel/cluster formation in sodium silicate systems across a range of concentrations. For this, they employed a model on a cubic lattice consisting of 125,000 sites in the canonical ensemble (NVT) with periodic boundary conditions, where Monte Carlo moves and minimization of the total system energy computed the structure evolution due to polymerization reactions. The energy calculation is based on the Gibbs free energy inputs for the dimerization reactions obtained by DFT [22,23,24] and molecular dynamic simulation computational approaches [25,26,27,28]. Later, in another study, White [9] developed the previous investigations further to explain zeolite and aluminosilicate gel formation quantitatively (CGMC simulations for metakaolin-based geopolymer systems), including a description of the impact of silicate/aluminosilicate precursor dissolution at the beginning of the process, and replicating the effects of elevated temperature and subsequent crystal nucleation. Moreover, Yang and White [20] used alkali-activated materials (AAMs), namely, metakaolin and class F fly ash, at the mesoscale through the CGMC modeling technique coupled with the DFT computational method. They reported that for both H-activated metakaolin and fly ash systems, the gel growth occurs through the formation of intermediate-sized clusters in conjunction with the growth of the largest particle. At the end of this process, some small clusters remain in the pore solution of the hardened gel, and their sizes depend on the degree of saturation of the pre-dissolved silicate concentrations in the system.

At the atomistic level, silicates and aluminosilicates have been investigated extensively. Simulations of aluminosilicate dissolution and geopolymerization have been performed using kinetic models [29]. Simulating silica sol–gel chemistry has been carried out using dynamic Monte Carlo simulations using reaction rates. For geopolymerization, dynamic Monte Carlo modeling requires in-depth knowledge of all atomistic processes, which is not currently available [30], where coarse-grained Monte Carlo modeling (CGMC) is an alternative method [31]. Micro-scale modeling of porous media is based on porous network modeling. Network models that represent the void space of a rock by a lattice of pores connected by throats can predict relative permeability once the pore geometry and wettability are known [32]. After assuming pores to be spherical, a clarified pore network model can be generated to reduce the computational effort. By transforming images with the city-block distance transform function and watershed algorithm, pore bodies and throats can be differentiated [33].

The main objective of the present work is to implement a 3D off-lattice Monte Carlo simulation of a coarse-grained model for studying the nucleation of alkaline aluminosilicate gel for the silicate-activated system. Implementing the tetrahedral structure as a constraint in the particle movement represents the main methodological novelty of the current paper. To implement an off-lattice coarse-grained Monte Carlo (CGMC) approach for the metakaolinite-based geopolymer, the Gibbs free energy of dimerization reactions are used here, extracted from the results reported by White et al. [9]. Therefore, four different monomer species of Si(OH)_4_, Al(OH)_4_^−^Na^+^, SiO(OH)_3_^−^·Na^+^·3H_2_O, and SiO_2_(OH)_2_^2−^·2Na^+^·6H_2_O are coarse-grained as particles, where each species is represented as one type particle, to compute the gel structure evolution as a function of the different number of iterations. The system’s total energy is also computed as a function of the different number of iterations. Moreover, the evolution trend for the computation of the cluster formation and metakaolinite dissolution were computed during 7 million iterations. The obtained structure was numerically characterized for cluster size and pore size distribution.

## 2. Simulation Model and Method

### 2.1. Atomistic Model Preparation

To implement the off-lattice CGMC approach, we started by defining the particle numbers for four different monomer species based on the selected activated solution and the pH. According to the selected silicate-activated system, the percentage of water as well as Na (68.4%), silicate in solution (10.6%), and metakaolin (21.0%) were extracted from the data published by White [9], shown in Table 1. For this, a cubic simulation box with a dimension of 200 Å was selected, and the percentage of each type of monomer species was computed. For the silicate-activated system, the contributions of silicate in the solution and metakaolin were equivalent to the simulation boxes with dimensions of 94.65 and 118.87 Å, respectively. Thus, the total number of silicate monomers in solution and metakaolin particles (in simplified crystal arrangement) in the simulation box with the average diameters of 7 and 5 Å was calculated as 2197 and 12,167, respectively. As there were three types of silicate monomers in solution, namely, Si(OH)_4_, SiO(OH)_3_^−^·Na^+^·3H_2_O, and SiO_2_(OH)_2_^2−^·2Na^+^·6H_2_O, the percentage of all three contributed silicate types (of 2197 particles in total), in order to keep the pH at 11, were computed to be 5%, 90%, and 5%, according to Figure 5, published by Šefčík and McCormick [34], which equals 110, 1977, and 110 particle numbers, respectively. Moreover, the percentage of particles in metakaolin existing as aluminate and silicate are 42% and 58%, respectively.

### 2.2. Monte Carlo Approach: Implementation in MATLAB Code

A MATLAB code was developed to implement the off-lattice CGMC approach through the consideration of monomer species as particles with different diameters (also representing different types of species) [35,36]. The condensation (polymerization) reaction was represented by binding the particles, where the system’s total energy was minimized. For this, the Gibbs free energy of dimerization reactions for the four different monomer species were used as the input table, taken from the literature [9] (obtained from the density functional theory (DFT) modeling method); for the off-lattice CGMC, the following procedures were adopted: The temperature and pressure were, respectively, 20 °C and 1 atm.

(1) First, we needed to simulate a silicate solution system that contained three types of dissolved silicate monomers (as particle types): Si(OH)_4_, SiO(OH)_3_^−^·Na^+^·3H_2_O, and SiO_2_(OH)_2_^2−^·2Na^+^·6H_2_O. Particles were subjected to pre-equilibration of the energy of the system for 1 million iterations, where only MC moves involving particles not contained in the metakaolin system were allowed to be accepted. The particle movement through the MC approach was accepted if the system’s total energy was lower than the former system before the movement. On the contrary, if the system’s total energy was higher (less negative) than the former system before the particle movement, a movement for each iteration may have been accepted if the probability of *X* was higher than a selected random number between 0 and 1. The probability of *X* was computed based on the Boltzmann factor associated with the configurational change as described by Equation (1), where *k_B_* is the Boltzmann constant, *T* is the temperature, and Δ*E* is the change in energy.
  (1) $$X=e{\;}^{\frac{-\Delta E}{kB\;T}}$$
  (2) $$\Delta E=E\;\left(after\;particle\;movement\right)-E\;\left(before\;particle\;movement\right)\;$$
  The dissolved three types of silicate particles in the solution were subjected to reaching the equilibrium condition (for our particular case, it took 1,000,000 iterations). Each iteration represented one random selection of a particle, including its movement to any arbitrary direction for a displacement, ensuring the particle connections always satisfied the tetrahedral structure. The displacement was generated by taking a random number from zero to one and multiplying it by the radius of a moving particle, and consequently determining whether the overlapping happened with the other particles. If yes, the particle went back to its previous position, and the next simulation loop was initiated. Finally, particles connected (into a cluster) when the distance between the moved particle and its nearest neighbor was within 1 Å; then, we calculated the increment/decrement of the system’s total energy [37]. Moreover, a tetrahedral geometry of the binding was respected, as described later (after Point 5 below).
(2) Once the system’s total energy did not change, the solution reached equilibrium. The metakaolin sub-system was involved in the MC particle selection and movement process. Thus, the metakaolin sub-system’s random dissolution occurred from the outer surfaces exposed to the solution. After picking one surface particle from the metakaolin sub-system and dissolving it into the solution, the rejection and acceptance were checked. The rejection was regarded as the case of overlapping of dissolved metakaolin particles with other particles in the solution and an update of the total energy of the system based on the probability of X as mentioned in Equation (1). It is also worth mentioning that in the case of silicate being selected from the surface of the metakaolin sub-system, the silicate species type was also required to be specified, whose selection was based on the maintenance of the equilibration requirement. Then, another iteration process was carried out with the dissolved particles in the solution to perform MC movement for polymerization (i.e., particle binding/clustering).
(3) It was essential to update the inner sites of the metakaolin system to become a part of the outer sites after each dissolution (particle removal) from the metakaolin system (after each dissolution iteration) and then continue the dissolution of metakaolin. Each dissolution process was followed by 30 MC iterations (i.e., particle movements) in the solution (Step 2).
(4) Steps 2, 3, and 4 were carried out until the end of metakaolin dissolution, or until it could not be dissolved anymore.
(5) After the program finished, the global scan method was used to output the cluster size distribution. Next, a clarified pore network model was generated by assuming pores to be spherical. Then, a watershed algorithm and city-block distance transform function were used for digitizing the particle structure, and throats and pore size distribution were deduced.

The realization time of the program involving the movements of the monomers and the metakaolin particle was approximately thirty-four hours. It was divided into twenty-four hours of MC simulation processing and eight hours of post-processing for the formation of the 3D graphics. The second part of the program, in which the pore and grain size were analyzed, lasted for two hours. All of this was performed using a computer: AMD Ryzen 7 PRO 4750U with Radeon Graphics 1.70 GHz-RAM: 16 GB. For handling a more extensive number of particles in a simulation system, the code was parallelized for use via a high performance computer.

**Figure 1:**
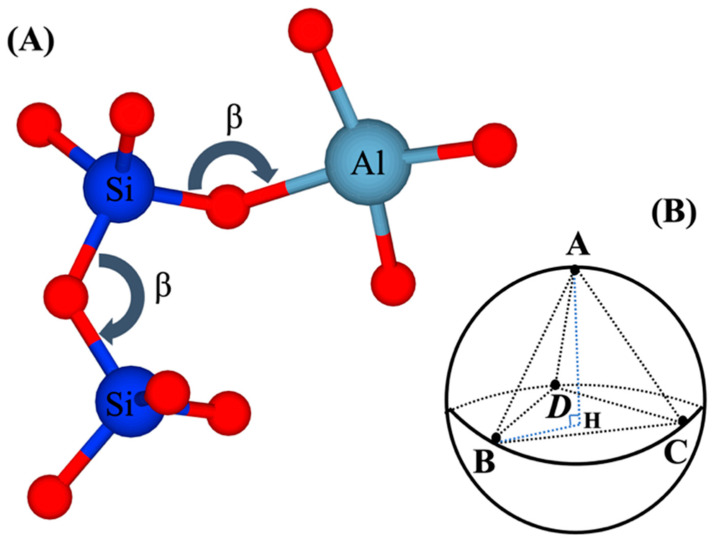
(**A**) Formation of Si-O-Si and Si-O-Al bonds which respect the β angle and tetrahedral geometrical constraints. (**B**) Tetrahedron constraints are expressed with four points ABCD on the unit sphere (coarse-grained particle) during the binding (particle association/aggregation) process.

According to the tetrahedra configuration of silicate and aluminate, the nucleation process takes place based on the tetrahedron formation distinguished with cartesian coordinates of ABCD, as defined on the four vertices of tetrahedra containing four faces and six edges on the unit sphere, as shown in Figure 1. The angle of β is defined as the rotational angle for dimerization formation (when the probability of dimerization reaction for two neighbor particles is accepted). Consequently, the two vertices must be rotated to form a bond.

## 3. Results and Discussion

**Figure 2:**
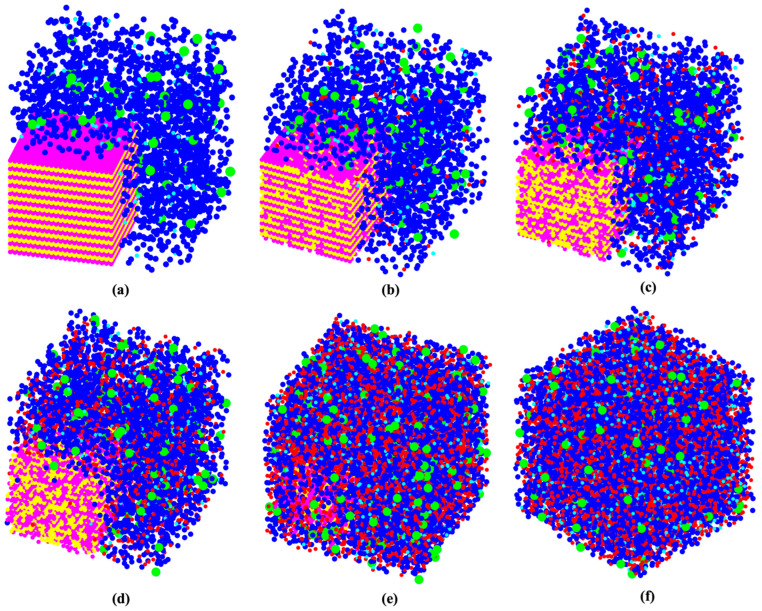
(**a**–**f**) The evolution of the six three-dimensional snapshots of the structures and cluster formations for the geopolymer extracted at a certain number of iterations of 0, 1000, 5000, 10,000, 100,000, and 7,000,000, respectively. The different monomer building units (coarse grained particles) are depicted with the following color codes: Si(OH)_4_ are given in cyan; SiO(OH)_3_^−^·Na^+^·3H_2_O, in blue; SiO_2_(OH)_2_^−^·2Na^+^·6H_2_O, in green; Al(OH)_4_^−^Na^+^, in red. A close-up view of Figure 2a, representing the solid metakaolinite phase (aluminate as yellow and silicate as magenta) is given in the Appendix A (Figure A1).

**Figure 3:**
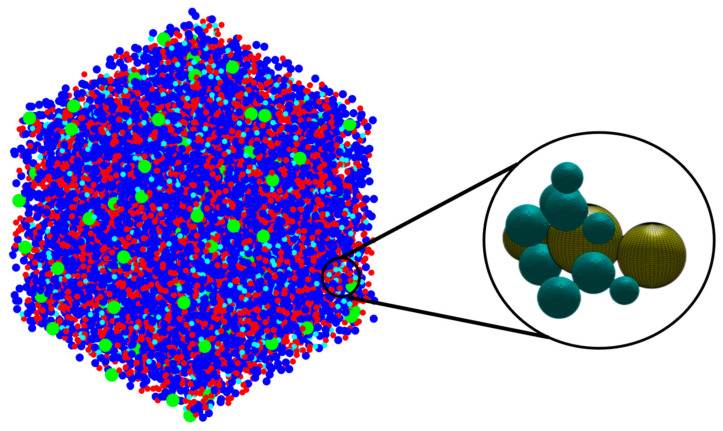
The final status of the simulation: The particles, clusters, and pore distribution are on the left side. Details are illustrated on the right side. Local details about the porosities and the distribution are shown on the right: the dark aqua marine colored particles represent the four different types of monomer building units (no distinction is made between their colors), and the yellow particles represent the pore sizes.

**Figure A1:**
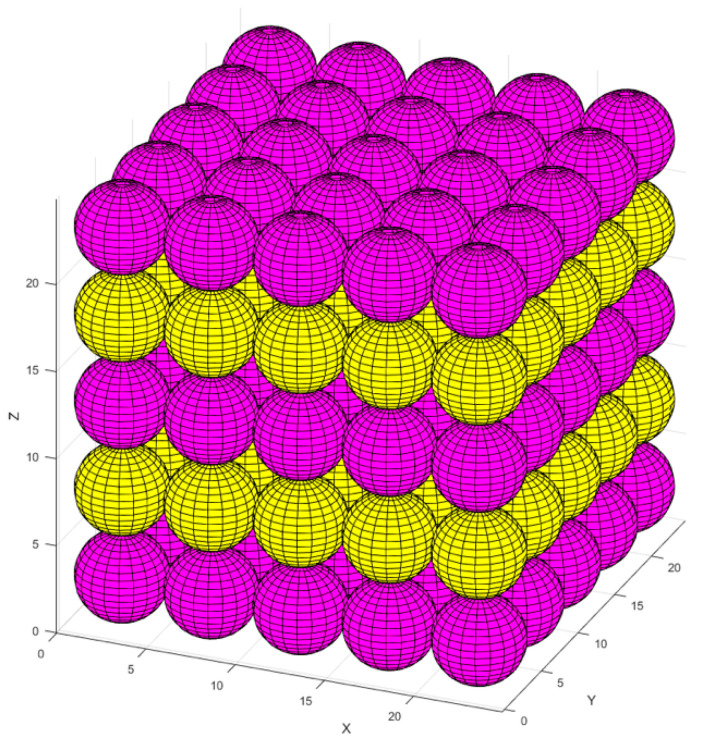
A close-up view of Figure 2a, representing the solid metakaolinite phase (aluminate units as yellow and silicate units as magenta).

Figure 2 has been plotted to illustrate the three-dimensional snapshots of the cluster formation of the geopolymer during the 7 million iterations as a simulation process. Figure 2a shows the initial structure at zero iteration and after 1 million pre-equilibrations, in which 91.80% of the total number of particles participated in the cluster formation. As the total metakaolinite (the close-up view of the solid metakaolinite phase has been illustrated in Figure A1 in the Appendix A) particles are dissolved at the iteration of 164,400, Figure 2d shows that some of the metakaolin particles remained and were not dissolved at the iteration of 100,000; in contrast, Figure 2e displays that the total metakaolinite particles were completely dissolved at the iteration of 400,000. Finally, Figure 2f shows the equilibrium condition, containing 66.50% of participated particles in the cluster formation. Figure 3 shows the initial and final simulation status of particles, clusters, pore distribution, and their different structures. In the zoomed-in detailed figure about the structure and pore distribution, the blue spherical shapes mean the different kinds of particles, and the yellow spherical shapes represent the different pores.

**Figure 4:**
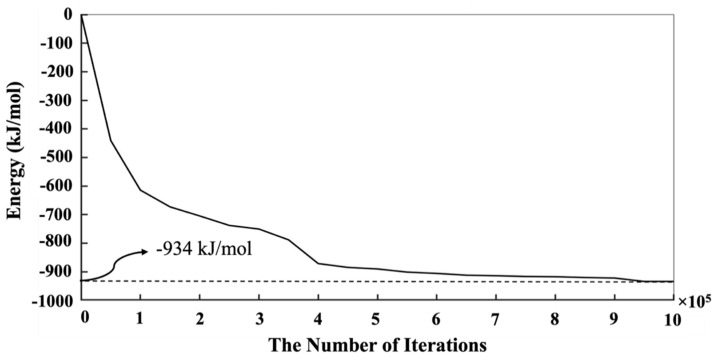
The energy evolution of the system to reach the equilibrium condition to obtain pre-condensation of the silicate solution (after 1 million iterations), where metakaolinite is not yet involved.

**Figure 5:**
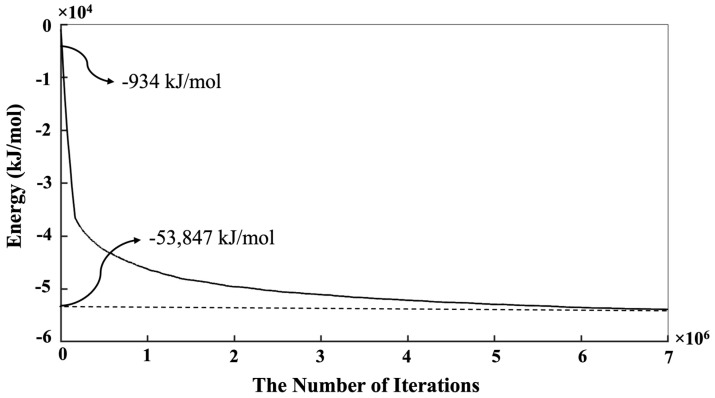
The equilibrium condition was obtained for the energy minimization computation of the silicate particles (taken as starting at 0 iterations) and metakaolinite for 7 million iterations. The point at which the metakaolin is “added” to the system is denoted as iteration 0. Thus, pre-equilibration takes place for an additional ‘‘1,000,000’’ iterations (shown in Figure 4).

**Figure 6:**
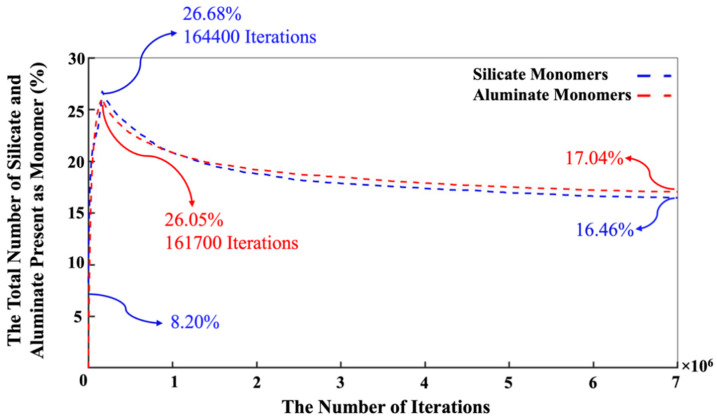
The change in the number of silicate and aluminate monomers present in the system during 7 million iterations. Metakaolinite particles are considered as monomers only when dissolved according to the dissolution process.

Figure 4 shows the equilibrium condition obtained for the energy computation of the silicate-activated system after 1 million iterations in the solution, where metakaolinite is not involved. As can be observed from Figure 4, the system’s total energy is zero at the beginning of the simulation. The system’s total energy is calculated from the summation of bonding energies of dimerized particles based on input parameters for the bonding energies between different particles, taken from White et al. [9]. By increasing the number of iterations, the system’s total energy is optimized (becomes more negative) due to cluster formation. Consequently, after 1 million iterations, the energy convergence occurred. In other words, there is no significant change in the system’s total energy after 1 million iterations, indicating that the system’s energy reached the equilibrium condition (of −934 kJ/mol, a case-specific absolute value that depends on system size). Once the solution reached the equilibrium condition after 1,000,000 iterations, the metakaolinite particle was allowed to participate in the process, and geopolymerization commenced. According to Figure 5, the point at which the metakaolin is ‘‘added’’ to the system is denoted at iteration 0, with pre-equilibration taking place from iteration ‘‘1,000,000’’ to iteration 0. To obtain the equilibrium condition after involving metakaolinite particles in the solution, at least 7 million iterations had to be performed. It can be observed that until the metakaolin is completely dissolved, the energy decreases rapidly, meaning the total energy of the system for the complete dissolution of metakolinite was obtained (−36,511.5 kJ/mol) at 164,400 iterations. After that and before 4,000,000 iterations, the total energy of the system decreased more gradually due to the condensation/aggregation process, with an increasing possibility to overlap and more limited Brownian motion, as a result, with a smaller possibility to find the correct particle for aggregation. From 4,000,000 to 7,000,000,000, the system’s total energy trend started reaching a steady state, meaning that all particles reached the equilibrium condition with total energy of −53,847 kJ/mol. Figure 6 has been plotted to illustrate the evolution trend for the existing two types of silicate and aluminate monomers in the system during 7 million iterations. At the beginning of the simulation, the contribution of aluminate monomers is zero; this is because all of the aluminate particles are presented in the metakaolinite.

**Figure 7:**
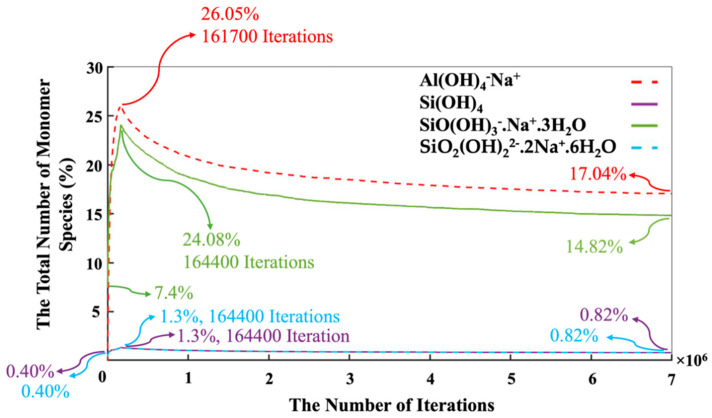
The evolution of the number of monomers (three silicates and one aluminate species type) present in the system during 7 million iterations. Metakaolinite particles were not considered at the beginning of the simulation (at zero iteration), as they were along the dissolution process.

**Figure 8:**
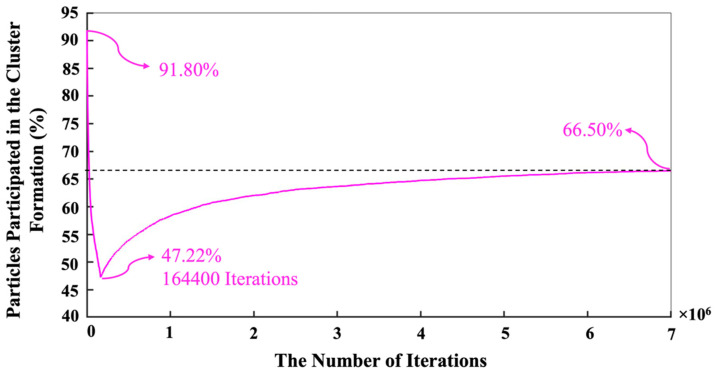
The evolution of the cluster formation in the system during 7 million iterations. The equilibrium condition for the cluster formation was obtained at a percentage of 66.35 after 7 million iterations. Metakaolinite particles were not considered at the beginning of the simulation (at zero iteration).

**Figure 9:**
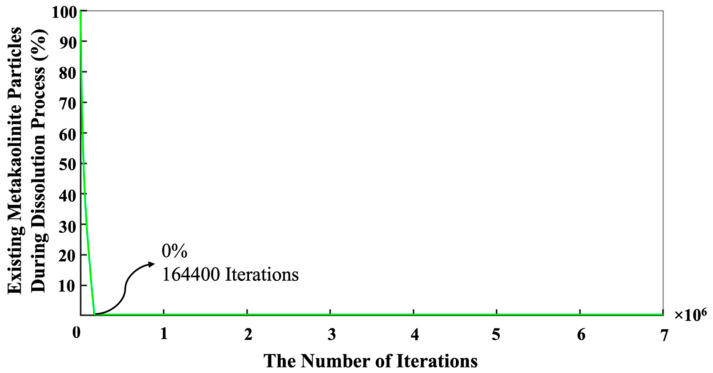
Dissolving process of metakaolinite during 7 million iterations. The total metakaolinite was dissolved after 164,400 iterations.

**Figure 10:**
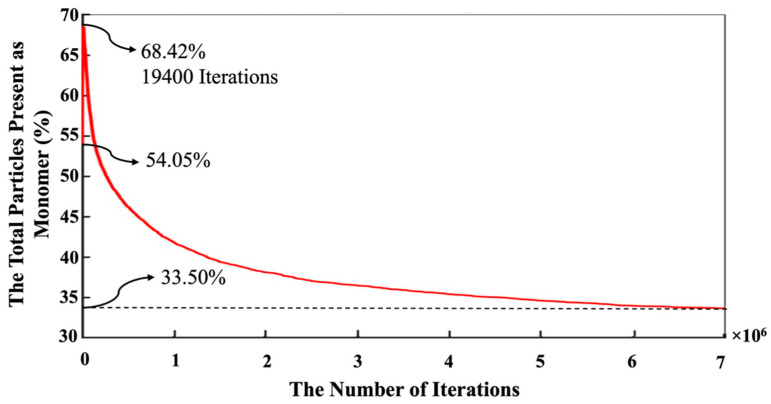
The number of the evolution of monomers in the system during 7 million iterations. The equilibrium condition for the existing monomers was obtained at a percentage of 33.65 after 7 million iterations. Metakaolinite particles were considered (as monomers) at the beginning of the simulation (at zero iteration).

**Figure 11:**
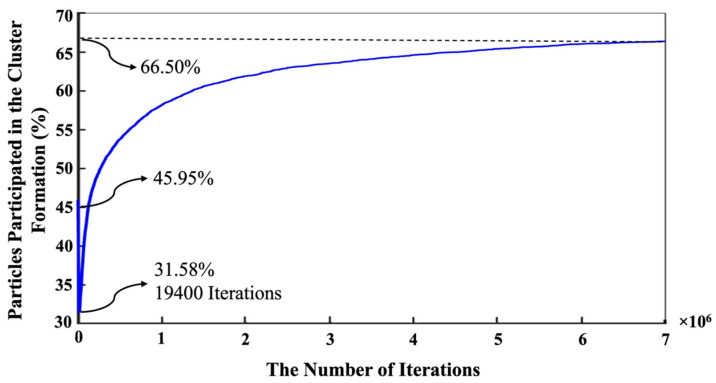
Number evolution of particles in the cluster formation during 7 million iterations. The equilibrium condition for the cluster formation was obtained as a percentage of 66.35 after 7 million iterations. Metakaolinite particles were considered as monomers at the beginning of the simulation (at zero iteration).

In contrast, the silicate monomers started with 8.20%, representing those that remained in the pre-condensed silicate solution, which was yet to participate in the binding/aggregation process during the initial first million pre-equilibrations. Then, the amount of silicate and aluminate monomers experienced a rapid increase (steep linear behavior) during the metakaolinite dissolution. The highest amount of the existing silicate and aluminate monomers in the system were observed at the iterations of 164,400 (the total metakaolinte is dissolved at this iteration) and 161,700, which equal 26.68% and 26.05%, respectively. At the iteration of 1,320,800, the proportion of aluminum monomers (20.10%) excels the silicate monomers (19.96%), and this relative ratio remained until the end of the simulation. Then, it took about 7 million iterations for the silicate and aluminate monomers to reach the equilibrium condition, in amounts of 16.46% and 17.04%, respectively. For illustration in more detail, Figure 7 has been plotted to specify the precise amounts of the four types of existing monomer species in the system during the 7 million iterations. Since there is one type of aluminum monomer species (Al(OH)_4_^−^·Na^+^) in the system, which comes from metakaolinite dissolution, the amount of it equals zero at the beginning of the simulation, as also manifested in Figure 6. The three different types of monomer species, namely Si(OH)_4_, SiO(OH)_3_^−^·Na^+^·3H_2_O, and SiO_2_(OH)_2_^2−^·2Na^+^·6H_2_O contributed with the amounts of 0.40%, 7.40%, and 0.40% (total 8.20%, as illustrated in Figure 6), respectively. According to one type of aluminum monomer species in the system, the existing amount of aluminum monomer at the highest point and at the equilibrium condition was equal to 26.05% and 17.04% (as also illustrated in Figure 4), respectively. Concerning silicate monomers, the monomer species of SiO(OH)_3_^−^·Na^+^·3H_2_O remained higher with a percentage of 14.82 than the two other monomer species of Si(OH)_4_ (0.82%) and SiO_2_(OH)_2_^2−^·2Na^+^·6H_2_O (0.82%) at the equilibrium condition after 7 million iterations. The reason for the highest existence of this type of silicate monomer species at the equilibrium condition can be explained by the high contribution of this type of monomer species at the beginning of the simulation. Figure 8 was plotted to further understand the changes happening for the cluster formation in the system. At the beginning of the simulation and after 1 million pre-equilibrium iterations, 91.80% of the total number of particles participated in the cluster formation (metakaolinite monomers were not considered). It can also be seen from Figure 6 that the presence of the remaining monomers, which were not involved in the cluster formation, is about 8.20%. As the dissolution of metakaolinite is started, the percentage of particles in the system increases; therefore, the percentage of contributed particles in the cluster formation decreases drastically until all of the metakaolinite is dissolved. The lowest percentage of 47.22 was observed for the total number of particles that participated in the cluster formation at the end of metakaolinite dissolution after 164,400 iterations. After 7 million iterations and at the equilibrium condition, 66.50% of the total numbers of particles participated in the cluster formation (33.50% is presented as total monomers of silicate and aluminate, as shown in Figure 8). Figure 9 also displays that after 164,400 iterations, the total amount of metakaolinte was dissolved. Figure 10 and Figure 11 illustrate the percentage of the total number of particles that remained as a monomer and contributed to the cluster formation considering metakaolinite monomers, respectively. These two figures are complementary toward each other, as monomers (initially dissolving from the metakaolin crystal) plus aggregated particles (in oligomers) represent the total amount of particles. Considering the metakaolinite particles (monomers) at the beginning of the simulation (at zero iteration), the percentage of the monomer particles increases from 8.20% to 54.05% as shown in Figure 6 and Figure 10, respectively. On the contrary, the cluster formation at the beginning of the simulation decreases from 91.80% to 45.95% by considering metakaolinite particles (as monomers), as shown in Figure 8 and Figure 11, respectively. In fact, through consideration of the metakaolinite particles in the system (at zero iteration), the percentage of the existing monomers increases. The highest existing particles as monomers were observed after 19,400 iterations as 68.42%, as shown in Figure 10. Figure 8 and Figure 11 prove that after 7 million iterations and at the equilibrium condition, the identical percentage of 66.50 was computed for the total number of particles that participated in the cluster formation. To investigate the simulation box effects with the different dimensions, all of the computations were also carried out for a cubic simulation box with the dimension of 500 Å, proving identical observations for the results.

**Figure 12:**
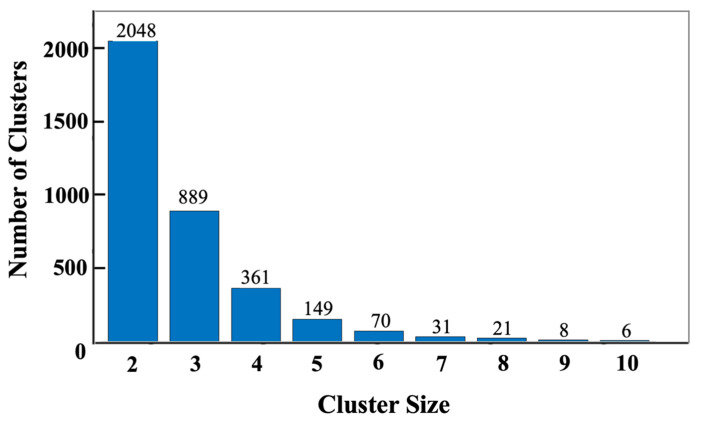
The cluster sizes (in terms of the number of single particles contained in the cluster) and their corresponding numbers after 7 million iterations.

When the simulation was completed, i.e., when the system’s energy reached its minimum, the cluster size present in the whole system was scanned. The example sizes (in terms of the number of single particles contained in the cluster) and their corresponding numbers are shown in Figure 12. When the system reaches equilibrium, 3583 clusters are generated, of which dimers and trimers occupy the absolute majority, 57% and 25%, respectively. As the size of the cluster increases, i.e., the number of single particles is increased, the proportion decreases until it reaches a maximum of 10 single particles. The particle–cluster aggregation model is adopted; so, the proportion of large-sized clusters is low.

**Figure 13:**
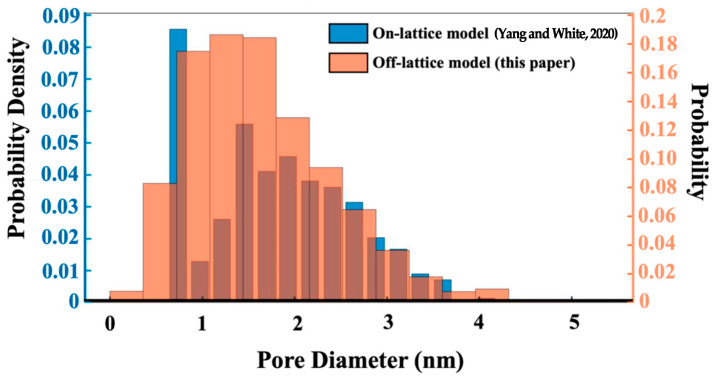
Cluster size distribution from the off-lattice model compared to the on-lattice results from Yang and White’s model [19] after 7 million iterations.

Figure 13 illustrates the pore size distribution. The pore size contains nano porous regions from 1 to 5 nm, and the most available distribution of pores is at 1.4 nm, with 1.05, 1.4, and 1.75 nm pores all occupying a relatively large probability. Before the binarization process, monomers and dimers are considered as aqueous species, i.e., part of the solution phase, following White et al. [19]. The solid at the beginning (if all are dissolved as monomeric particles) is 17.1% of the volume percentage of the whole system. At the end of the simulation, the cluster solid phase takes 6.9% of the volume percentage of the whole system, and all of the clusters with their intra-cluster formed porosity is 11.8%. So, the volume fraction of the cluster is expanded by 71.0% due to the packing density of the clusters. The solid at the end of the simulation (all of the clusters and their formed porosity) is 12% of the volume percentage of the whole system. In addition, the simulation results of this model are compared with those of Yang et al. and show three differences: 1. the pore size distribution is slightly wider, especially around 4 and 5 nm; 2. more pore sizes in this model are concentrated between 1 and 2 nm; and 3. compared with the results of Yang et al., a significant portion of pore sizes are concentrated at 0.8 nm. The pore size distribution of the present model is closer to the Gaussian distribution and is more consistent with the actual experimental results measured by Yang et al. (Figure 10 in Yang’s paper), indicating that the off-lattice model is closer to the experimental test results and more accurately demonstrates the real structural characteristics of microscopic dimensions than the on-lattice model.

## 4. Conclusions

This paper’s novelty is developing the 3D off-lattice coarse-grained Monte Carlo (CGMC) approach to study the nucleation of alkaline aluminosilicate gels. Compared to the previous on-lattice approach by White et al. (2012), which is computationally much easier to implement, the off-lattice approach can consider tetrahedral geometrical constraints when binding the monomer particles into aggregated clusters (Figure 1). Thus, our model adopts the more advanced collision theory of particle–cluster aggregation.

The results of the cluster size distribution show that the largest cluster contains ten particles, while the clusters of dimers and trimers dominate the distribution at, respectively, 57% and 25%. In future work, a cluster–cluster collision model, which can allow the movement of clusters and the aggregation with other clusters, can be chosen to explore its effect on the cluster size and pore size distribution.

The off-lattice-based MC simulation process gives more degrees of freedom for the movement of particles and clusters. This enables one to obtain more consistent final virtual nanostructures of the agglomeration for better agreement with the actual situation. Moreover, as the model’s driving rules for aggregating coarse-grained monomers are based on energy and geometrical considerations, it has broader applicability to be easily adjusted to the reaction material, temperature, and pressure conditions. Compared with the on-lattice model, the off-lattice theory used in this model exhibits a pore size distribution on the nano scale, which is in better agreement with the Gaussian distribution and the measurement results. This demonstrates the superiority of the off-lattice model in the study of nano size gel structure characteristics. A more accurate description of the virtual nanostructures is a crucial step toward a multi-scale simulation for relating the structure with materials’ properties. For example, this is needed for chemical reactivity and rheological, mechanical, and transport properties relevant for geopolymer binder applications in concrete structures, where workability/formability, setting/hardening time, and durability, respectively, play a key role.

## Figures and Tables

**Table 1:** Gibbs free energy of dimerization reactions ($\Delta G_{reaction};\;\mathrm{kJ}/\mathrm{mol}$) occurring in an aluminosilicate solution at a pH of 11 [9].

Monomer Species	M	M^−^·Na·3H_2_O	M^2−^·2Na·6H_2_O	A^−^·Na
M	−1.8	−9.3	−5.3	−21.2
M^−^·Na·3H_2_O		−0.9	8.1	−9.7
M^2−^·2Na·6H_2_O			35.0	14.5
A^−^·Na				16.9

M = Si(OH)_4_, M^−^ = SiO(OH)_3_*^−^*, M^2−^ = SiO_2_(OH)_2_^2*−*^, A^−^ = Al(OH)_4_^−^.

## Data Availability

The data presented in this study are available upon request from the corresponding author.
